# “The least significant change on bone mineral density scan increased in patients with higher degrees of obesity”

**DOI:** 10.1007/s40520-024-02736-4

**Published:** 2024-04-23

**Authors:** Claire Dorilleau, Lukshe Kanagaratnam, Isabelle Charlot, Ambre Hittinger, Eric Bertin, Jean-Hugues Salmon, Marion Geoffroy

**Affiliations:** 1 Rheumatology Department, University Hospital Center of Reims, 45 Rue Cognacq-Jay, Reims, Reims, 51092 France; 2 Department of Clinical Research and Innovation, University Hospital Center of Reims, Reims, France; 3 Performance, Health, Metrology, Society Laboratory (PSMS, EA 7507) of Reims Champagne-Ardenne University and Clinical Nutrition Transversal Unit (UTNC) of Reims University Hospital, Endocrinology Nutrition Department, University Hospital Center of Reims, Reims, France; 4 Faculty of Medicine, URCA - University of Reims Champagne Ardenne, Reims, France

**Keywords:** DXA, Obesity, Least significant change, Osteoporosis, Bone mineral density

## Abstract

**Background**: The least significant change (LSC) threshold of 0.03 g/cm² is used to interpret bone mineral density (BMD) scans in the general population. Our working hypothesis was that the current LSC threshold would not be applicable in obese populations. **Aims**: The aim of this study was to calculate the LSC in an obese population. **Methods**: We performed an interventional study among 120 obesity patients, in whom two measurements of BMD were performed at 3 sites. Pairs of measures were used to calculate the LSC, using the Bland and Altman method. **Results**: We calculated that the LSC was 0.046 g/cm² at the lumbar spine, 0.069 g/cm² at the femoral neck, and 0.06 g/cm² at the total hip. We also calculated the LSC for each class of obesity and observed an increase in LSC with increasing body mass index (BMI). We calculated a LSC of 0.05 g/cm² in patients with class 2 or class 3 obesity, whereas the LSC in patients with class 1 obesity is similar to the threshold used in the general population. **Discussion**: In obese population, like BMD, LSC is higher than the threshold value of the general population, and increases with increasing BMI.**Conclusion**: LSC of 0.05 g/cm² could be used in clinical practice in patients with class 2 or 3 obesity. These findings should help to improve the interpretation of BMD scans in these patients and optimize their management. **Trial registration number**: Comité de Protection des Personnes Ile-de France VII, France.

## Introduction

In subjects with obesity, a chronic disease defined as a body mass index (BMI) ≥ 30 kg/m² according to the French national health authority [1], metabolism, bone pathophysiology and risk of fracture undergo changes [2, 3]. Indeed, several studies have shown an increase in BMD in populations with obesity, although this increase was not found to protect them against a risk of fracture [3–6]. According to the World Health Organization (WHO), the number of people with obesity worldwide has doubled since 1980, became a public health issue [1]. Consequently, physicians are increasingly encountering patients in their daily practice who meet the criteria for obesity [7].

Dual-energy X-ray absorptiometry (DXA) scan is the standard technique for the measurement of bone mineral density (BMD), in the aim of detecting or monitoring osteoporosis and adapting therapeutic management [8–11]. For comparing repeated DXA scans, the least significant change is calculated, which is the smallest change in BMD that is considered to be statistically significant. In the general population, the LSC is 0.03 g/cm² [11], i.e. BMD loss is considered significant if the variation in BMD is greater than 0.03 g/cm² between two scans in the same patient. The LSC makes it possible to follow the evolution of bone density between two scans, and to adapt the patient’s therapeutic management accordingly.

In view of the rising incidence of obesity worldwide, and the specificities of populations with obesity in terms of BMD, we sought to investigate whether current methods for investigating and monitoring BMD, namely a threshold for LSC of 0.03 g/cm², was appropriate for the interpretation of BMD scans in populations with obesity, given that they have a higher BMD at the outset. We hypothesized that this study could provide useful guidance for the monitoring of BMD in populations with obesity, thereby helping to optimize their management. Our working hypothesis was that the current threshold for LSC, namely 0.03 g/cm², would not be applicable in populations with obesity. The aim of this study was to calculate the LSC in a population with obesity. The secondary objective was to calculate the LSC for each class of obesity.

## Methods

### Study design

We performed a single-centre, interventional study that included 120 patients who underwent BMD evaluation by DXA scan in the Rheumatology department of our medical center, between November 2019 and July 2020, and who had a BMI ≥ 30 kg/m². All subjects had to be aged 18 years or older, have social security coverage, and provide written informed consent before they could be included. We excluded those aged under 18 years, subjects under legal or judicial protection, and pregnant women. Included subjects were divided into three groups according to the class of obesity (class 1, 2 or 3). Class 1 obesity was defined as a BMI between 30 and 35 kg/m², class 2 as a BMI between 35 and 40 kg/m² and class 3 (or morbid obesity) as a BMI ≥ 40 kg/m².

### Protocol

According to Roux and al [12]. , a minimum of 30 patients must be included to enable calculation of the LSC. Each subject has to have two measures of BMD at 3 different sites, namely the lumbar spine (L1-L4), the femoral neck and the total hip. Measures of BMD were obtained by dual-energy X-ray absorptiometry (DXA) scan, and expressed in g/cm².

The least significant change (LSC) represents the smallest difference between successive measurements of bone mineral density (BMD) that can be considered to be a real change and not attributable to chance. This is a method to determine if a difference between two successive measurements is statistically significant. It provides a threshold value for a significant difference, distinct from p-values. Unlike traditional hypothesis tests that use a p-value to determine statistical significance, the LSC is based on the variation of measurements within an individual. Thereby, no group control was planned in the protocol study.

In all patients, BMD was measured twice, at least of the 3 sites. Standard procedure in our institution for DXA scan acquisition did not include manually holding back the fat pad of the belly (panniculus) if it was overlying the proximal femur, during measurements of the total hip. It is difficult to hold back the fat pad manually, either by the patient who, depending on his BMI and functional capacity, cannot lift his own fat pad during the DXA scan, furthermore it is also possible that anatomical alterations occur in the positioning of obese patient after fat layering (e.g. introducing lumbar lordosis) and thus alter the DXA measurements, nor by the trained operator due to the unnecessary risk of radiation exposure during the DXA scan. We performed a first DXA scan, then the patient was asked to get up off the examination table and stand up. Then, the patient was put in position again for the second measurement. All pairs of measures were performed on the same day, and the pairs of measures were used to calculate the LSC [12, 13].

We limited measurement bias on the BMD scans since all scans were performed by two trained operators with expertise in DXA scans [14]. Furthermore, all DXA scans were performed on the same machine (HOLOGIC Discovery) [15], and the two measurements were performed on the same day in each patient. Quality control of reproducibility was performed daily, using a Hologic anthropomorphic phantom.

### Data recorded

Each patient was questioned about the presence of possible risk factors for osteoporosis. We recorded age, sex, weight, height and ethnicity for all patients [16]. We also recorded BMD, T-score, Z-score at each measurement site.

### Statistical analysis

The size of the sample are calculated according to the recommendations described by Roux and Ravaudand al [12] (13). In our study, a total of 120 patients were included to enable subgroup analysis for each class of obesity, at least 30 patients par subgroup.

Quantitative data are described as mean ± standard deviation, and qualitative variables as number (percentage). The Bland and Altman method was used to calculate the LSC. The limits of agreement were calculated according to the following equation: d ± z ( 1 – α / 2 )SD diff, where d is the mean of the differences, and SD diff is the standard deviation of the differences, and z (1 – α / 2 ) is equal to 100( 1 – α / 2)^th^ percentile of the normal distribution. The expected value of d is zero, since it is considered that there should be no real difference in BMD between two measures performed on the same day in the same subject. Considering α equal to 5%, the equation is equivalent to ± 1.96 SD diff. This formula can also be expressed as ± 1.96 √2 SD where SD is intra-subject variability [13, 17, 18].

### Ethical considerations

Informed consent was obtained from all individual participants included in the study. Patients were informed about the objectives of the study by the physician prescribing the DXA scan, and were informed about the study again on the day of the examination by the investigator. An information leaflet was given to each participant, and they were required to provide written informed consent before the scans. Patients were informed about the exposure to radiation during the DXA scan, which was evaluated at 1 to 15 µSv. By way of comparison, natural exposure to radiation is estimated to be 6 µSv per day [19, 20]. All procedures performed in studies involving human participants were in accordance with the ethical standards of the institutional and/or national research committee and with the 1964 Helsinki declaration and its later amendments or comparable ethical standards. This study was approved on 09 October 2019 by the Ethics Committee.

## Results

### Patient characteristics

In total, 120 patients were included in the study. The characteristics of the population are displayed in Table 1.

**Table 1:** Characteristics of the study population (*N* = 120) overall, and according to class of obesity

	Overall *N* = 120	Class 1 obesity *N* = 44	Class 2 obesity *N* = 42	Class 3 obesity *N* = 34
Age (years)				
Mean ± standard deviation	53 ± 13	57 ± 12	53 ± 13	44 ± 12
Weight (kg)				
Mean ± standard deviation	102 ± 17.40	89 ± 9.80	100 ± 10.90	121 ± 15
Body mass index (kg/m²)				
Mean ± standard deviation	37.40 ± 5.10	32.45 ± 1.49	37.27 ± 1.63	44.03 ± 3.34
Sex:				
- Females, N (%) - Males, N (%)	81 (67.50%) 39 (32.50%)	28 (63,63%) 16 (36,36%)	30 (71,42%) 12 (28,57%)	23 (67,64%) 11 (32,35%)
Ethnicity:				
- Caucasian, N (%) - African, N (%)	119 (99.17%) 1 (0.83%)	44 (100%) 0 (0%)	42 (100%) 0 (0%)	33 (97,05%) 1 (2,94%)
Postmenopausal women (*N* = 81) N (%)	44 (53.66%)	22 (50,00%)	16 (36.36%)	6 (13.63%)
Rheumatic disease	24 (20,00%)	10 (22,72%)	9 (21,42%)	5 (14,70%)

Overall, 44 patients had class 1 obesity, 42 had class 2 and 34 had class 3 obesity. The average BMD values at the lumbar spine, femoral neck and left total hip, as well as the T and Z scores, are shown in Table 2.

**Table 2:** Bone mineral density in the study population

Population		Mean ± SD BMD (g/cm²)	T-score	Z-score
All *N* = 120	Lumbar spine Femoral neck Total hip	1.083 ± 0.152 0.868 ± 0.153 1.026 ± 0.157	0.420 ± 1.410 -0.100 ± 1.230 0.380 ± 1.050	0.970 ± 1.370 0.790 ± 1.000 0.940 ± 0.950
Class 1 obesity *N* = 44	Lumbar spine Femoral neck Total hip	1.057 ± 0.186 0.822 ± 0.139 0.973 ± 0.143	0.100 ± 1.650 -0.520 ± 1. 060 -0.060 ± 0.930	0.780 ± 1.650 0.550 ± 0.930 0.640 ± 0.850
Class 2 obesity *N* = 42	Lumbar spine Femoral neck Total hip	1.068 ± 0.123 0.839 ± 0.143 1.003 ± 0.154	0.350 ± 1.180 -0.310 ± 1.170 0.250 ± 1.070	0.880 ± 1.120 0.620 ± 0.940 0.830 ± 0.940
Class 3 obesity *N* = 34	Lumbar spine Femoral neck Total hip	1.135 ± 0.123 0.964 ± 0.143 1.124 ± 0.135	0.920 ± 1.20 0.700 ± 1.140 1.120 ± 0.960	1.320 ± 1.220 1.290 ± 1.000 1.470 ± 0.880

### Calculation of the LSC in a population with obesity

**Fig. 1:**
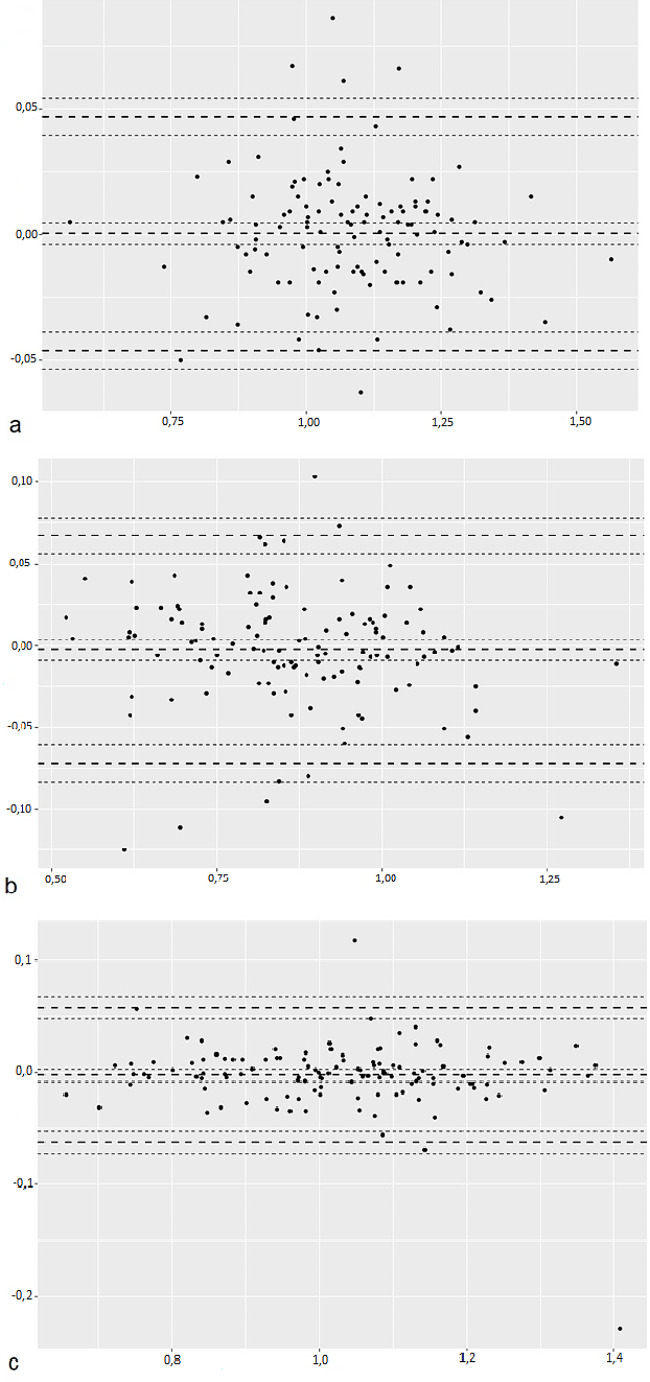
Bland and Altman plot of the mean bone mineral density (X-axis) against the difference between the 2 measurements of bone mineral density (Y-axis) at the lumbar spine (**a**), femoral neck (**b**) and total hip (**c**). The upper line (large dashes) represents the least significant change

The LSC was calculated at 0.046 g/cm² at the lumbar spine, 0.069 g/cm² at the femoral neck and 0.06 g/cm² at the total hip (Fig. 1., Table 3).

**Table 3:** Least significant change in the study population, by class of obesity

Population	Mean difference in BMD^a^ (g/cm²)	SD^b^	LSC^c^
Overall population *N* = 120			
- Lumbar spine - Femoral neck - Total hip	0.0001 -0.0027 -0.0027	0.0230 0.0350 0.0300	0.0465 0.0695 0.0600
Class 1 obesity *N* = 44			
- Lumbar spine - Femoral neck - Total hip	0.0005 -0.0024 -0.0003	0.0159 0.0358 0.0163	0.0312 0.0704 0.0320
Class 2 obesity *N* = 42			
- Lumbar spine - Femoral neck - Total hip	-0.0031 0.0002 -0.0031	0.0276 0.0351 0.0237	0.0542 0.0688 0.0465
Class 3 obesity *N* = 34			
- Lumbar spine - Femoral neck - Total hip	0.0038 -0.0064 -0.0059	0.0264 0.0361 0.0481	0.0527 0.0707 0.0943

a: BMD, bone mineral density ; b : SD, standard deviation ; c : LSC, least significant change

### Calculation of the LSC according to class of obesity 

In subjects with class 1, 2 and 3 obesity, the LSC was respectively calculated to be 0.031, 0.054 and 0.052 g/cm² at the lumbar spine; 0.070, 0.068 and 0.070 g/cm² at the femoral neck; and 0.032, 0.046 and 0.094 g/cm² at the total hip (Table 3).

## Discussion

This study calculated the least significant change on bone mineral density scans in a population of subjects with obesity. The LSC values in this population are different to the threshold applied in the general population. Indeed, we calculated a LSC of 0.046 g/cm² at the lumbar spine, 0.069 g/cm² at the femoral neck and 0.06 g/cm² at the total hip in this population of 120 patients with obesity.

Numerous studies have investigated the LSC on BMD scans, mainly in post-menopausal women [12, 18], young subjects, or those with chronic inflammatory rheumatic diseases [9, 21]. However, this is the first study to specifically investigate the LSC in patients with obesity. Indeed, we could not find any study in the literature that focused on the calculation of a specific LSC for populations with obesity. Yet, in daily practice, we increasingly encounter patients with obesity, and the specific management that this requires. It has been shown that BMD is higher in populations with obesity [2, 4], suggesting that the interpretation of BMD scans in populations with obesity may also be different.

The LSC for the general population is estimated at 0.034 g/cm² at the lumbar spine, 0.036 g/cm² at the femoral neck and 0.027 g/cm² at the total hip [18]. Various studies, including ours, have shown divergent results according to the measurement site [17, 21]. In practice, to simply the interpretation of the examination and to harmonize practices, a single value of 0.03 g/cm² is used in the general population for all sites of measurement (lumbar spine, femoral neck or total hip) of the BMD. However, using a single value to interpret BMD scans, instead of specific values for each measurement site, incurs a risk of under-estimation, or over-estimation of potential loss of BMD in a given patient, with the potential to impact on the therapeutic decisions [11].

Furthermore, our study suggests that an interpretation that takes account of the BMI of each patient would be more suitable, since the values calculated in our study differed across classes of obesity, and between measurement sites.

In practical terms, using a different threshold value for each measurement site, while also taking account of each individual’s BMI may appear substantially more complex for the physician, but is more tailored to the individual patient [22]. It would be necessary to define an LSC for each class of obesity for the interpretation of DXA results, in the same way as the threshold applied in the general population.

The results of this study show that DXA in patients with obesity should be interpreted carefully [23]. The results suggest that subjects with class 1 obesity have a LSC that is close to the value applied in the general population, and therefore, the interpretation of repeated BMD scans in these individuals need not be changed. However, for subjects with class 2 or 3 obesity, the LSC values calculated in our study are higher than the threshold used in the general population. A LSC of 0.05 g/cm² might be appropriate for subjects with stage 2 or 3 obesity, to define a significant change in BMD between two scans. The use of this new threshold might make it possible to not overtreat in certain people with obesity. It is important to underline that the LSC must be used for the evaluation of bone mineral density to adapt the patient’s treatment, but that the diagnostic of osteoporosis in patient with or without obesity is still based on the T-score (or Z-score). Also, in case of outlier results, we must consider controlling the DXA a second time.

The main strength of our study is the originality of the subject. Furthermore, we included a sufficient number of subjects to enable to calculation of the LSC for each class of obesity, with reliable results. A further strength is the very low number of missing data. In our study, we chose to calculate the LSC rather than the coefficient of variation [13, 17, 24], in order to ensure independence from the BMD value. Indeed, the coefficient of variation increases with decreasing BMD, thus overestimating the loss of BMD [25]. Consequently, the LSC appears to be more reliable, and seems to provide a result that is not impacted by the patient’s BMD [17, 22].

The limitations of our study are related to the difficulty of measuring BMD in subjects with morbid obesity [26]. Firstly, there are equipment limitation, because the maximum weight authorized varies between DXA machines, ranging from 160 to 205 kg [27, 28]. Secondly, it challenging to put the patient into position and to hold back the fat pad of the belly (panniculus) and keep it clear from the zone where the BMD of the femur is measured [27]. Thirdly, obese patient is very tick and highly attenuates the X-rays, resulting in poor image quality and absorptiometry statistics modifications [28]. In fact, these limitations are related to the interpretation, due to the reduction in photon penetration through soft tissue. A high proportion of fat mass may distort the interpretation of BMD [29, 30]. Measurement error in BMD using DXA scans increases with increasing BMI and weight [31, 32].

Fourthly, the uneven fat distribution and the variability composition of obese patient can also affect the accuracy of BMD measurements with varying proportions of adipose tissues, muscle and bone. This can make it difficult to differentiate soft tissues and bones, resulting in measurement errors [33].

All of these limitations could justified a closer control of DXA in the specific population. To limit this measurement bias, it would be interesting to perform whole-body DXA systematically coupled with BMD scan to investigate a possible relation between the results obtained in this study and the measure of the panniculus. To perform this measurement, we could use visceral adipose tissue [34] or the direct measure of the fat pad around the belly (abdominal circumference), and the peri-trochanteric region of the femur. Moreover, the analysis of microarchitecture with the Trabecular Bone Score (TBS) in addition to the measurement of LSC could also be a good way to improve the bone mineral density scans interpretation in a population of subjects with obesity [35].

## Conclusion

In conclusion, the LSC on BMD scan in populations with obesity in our study was found to be 0.046 g/cm² at the lumbar spine, 0.069 g/cm² at the femoral neck and 0.06 g/cm² at the total hip. These values are higher than the threshold commonly used in the general population, and is higher with increasing BMI. Based on these findings, a LSC of 0.05 g/cm² could be used in patients with class 2 or 3 obesity. Further studies are warranted with larger populations to compare these values and investigate their utility in daily practice. This could help to simplify interpretation, while providing more personalized follow-up for each patient.

## Data Availability

No datasets were generated or analysed during the current study.

## References

[1] World Health Organization (2017) Obesity and Overweight Factsheet No. 311

[2] Shapses SA, Pop LC, Wang Y (2017) Obesity is a concern for bone health with aging. Nutr Res N Y N. Mar:1–13. 10.1016/j.nutres.2016.12.010 10.1016/j.nutres.2016.12.010 PMC5385856 28385284

[3] Silvia Migliaccio EA, Greco R, Fornari LM, Donini LD, Luigi (2013) Andrea Lenzi. Skeletal alterations in women affected by obesity. *Aging Clin Exp Res*. Oct:25 Suppl 1:S35 7. 10.1007/s40520-013-0090-1 10.1007/s40520-013-0090-1 24061852

[4] Bachmann KN, Bruno AG, Bredella MA, Schorr M, Lawson EA, Gill CM (2016). Vertebral strength and estimated fracture risk across the BMI spectrum in women. J Bone Min Res.

[5] Krishnan C, Choksi P, Peterson MD (2017 Nov-Dec) Abdominal adiposity and low physical activity are independently and inversely associated with bone mineral density. Obes Res Clin Pract 11(6):740–746. 10.1016/j.orcp.2017.04.002Get rights and content 10.1016/j.orcp.2017.04.002 28416385

[6] Alsaed OS, Al-Allaf AW, Elgenaied I, Jebril RA, Sasi S, Ahmed AO (2021). Increased fracture risk after bariatric surgery: a case -controlled study with a longterm follow-up. Obes Surg.

[7] Leslie WD, Lix LM, Yogendran MS, Morin SN, Metge CJ, Majumdar SR (2014). Temporal trends in obesity, osteoporosis treatment, bone Mineral Density, and fracture rates: a Population-based historical cohort study. J Bone Min Res.

[8] Lees B, Stevenson JC (1992). An evaluation of dual-energy X-ray absorptiometry and comparison with dual-photon absorptiometry. Osteoporos Int.

[9] Alain Lescoat M, Leroy G, Coiffier C, Cazalets N, Belhomme (2021). Bone mineral density and trabecular bone score assessment in systemic sclerosis: a cross-sectional study. Joint Bone Spine.

[10] Carlene AJ, Stoklossa M, Forhan RS, Padwal MC, Gonzalez CM (2016). Prado. Practical considerations for body composition Assessment of adults with class II/III Obesity Using Bioelectrical Impedance Analysis or Dual-Energy X-Ray Absorptiometry. Curr Obes Rep.

[11] Briot K, Roux C, Thomas T, Blain H, Buchon D, Chapurlat R (2018). Actualisation 2018 des recommandations françaises du traitement de l’ostéoporose post-ménopausique. Rev Rhum oct.

[12] Roux C, Garnero P, Thomas T, Sabatier J-P, Orcel P, Audran M (2005). Recommendations for monitoring antiresorptive therapies in postmenopausal osteoporosis. Jt Bone Spine Rev Rhum Jan.

[13] Ravaud P, Reny JL, Giraudeau B, Porcher R, Dougados M, Roux C (1999). Individual smallest detectable difference in bone mineral density measurements. J Bone Min Res.

[14] Feit A, Levin N, McNamara EA, Sinha P, Whittaker LG, Malabanan AO (2020). Effect of positioning of the region of interest on bone density of the hip. J Clin Densitom sep.

[15] Tothill P, Fenner JAK, Reid DM (1995). Comparisons between three dual-energy X-ray absorptiometers used for measuring spine and femur. Br J Radiol Jun.

[16] Wu Q, Dai J (2023). Racial/Ethnic differences in bone Mineral density for osteoporosis. Rev Curr Osteoporos Rep.

[17] Lodder MC (2004). Reproducibility of bone mineral density measurement in daily practice. Ann Rheum Dis mar.

[18] Kolta S, Ravaud P, Fechtenbaum J, Dougados M, Roux C (2000). Follow-up of individual patients on two DXA Scanners of the same manufacturer. Osteoporos Int sep.

[19] Njeh CF, Fuerst T, Hans D, Blake GM, Genant HK (1999). Radiation exposure in bone mineral density assessment. Appl Radiat Isot Jan.

[20] ISCD (The International Society for Clinical Densitometry) Pediatric Resources [Internet]. [cité 19 avr 2019]. Disponible sur: https://www.iscd.org/patientinformation/bone-density-pediatric

[21] El Maghraoui A, Do Santos Zounon AA, Jroundi I, Nouijai A, Ghazi M, Achemlal L (2005). Reproducibility of bone mineral density measurements using dual X-ray absorptiometry in daily clinical practice. Osteoporos Int Dec.

[22] Nguyen TV, Eisman JA (2000). Assessment of significant change in BMD: a New Approach. J Bone Min Res.

[23] Lui C, Wu D, Zhang JF, Xu D, Xu WF, Chen Y (2016). Changes in bone metabolism in morbidly obese patients after bariatric surgery: a Meta-analysis. Obes Surg Jan.

[24] Glüer C-C, Blake G, Lu Y, Blunt BA, Jergas M, Genant HK (1995). Accurate assessment of precision errors: how to measure the reproducibility of bone densitometry techniques. Osteoporos Int Jul.

[25] Maggio D, McCloskey EV, Camilli L, Cenci S, Cherubini A, Kanis JA (1998). Short-term reproducibility of proximal femur bone Mineral Density in the Elderly. Calcif Tissue Int oct.

[26] Rothney MP, Brychta RJ, Schaefer EV, Chen KY, Skarulis MC (2009). Body composition measured by dual-energy X‐ray absorptiometryhalf‐body scans in obese adults. Obes (Silver Spring).

[27] Binkley N, Krueger D, Vallarta-Ast N An overlying fat panniculus affects femur bone mass measurement. J Clin Densitom 2003 Fall ;6(3):199–204. 10.1385/JCD:6:3:199 10.1385/jcd:6:3:199 14514987

[28] Dual energy x ray absorptiometry for (2010) bone mineral density and body composition assessment, iaea human health series no. 15, international atomic energy agency vienna

[29] Yu EW, Thomas BJ, Brown JK, Finkelstein JS (2012). Simulated increases inbody fat and errors in bone mineral density measurements by DXAand QCT. J Bone Min Res.

[30] Johnson Stoklossa CA, Forhan M, Padwal RS, Gonzalez MC, Prado CM (2016). Practical considerations for body composition Assessment of adults with class II/III Obesity Using Bioelectrical Impedance Analysis or Dual-Energy X-Ray Absorptiometry. Curr Obes Rep Dec.

[31] Knapp KM, Welsman JR, Hopkins SJ, Fogelman I, Blake GM (2012). Obesity increases Precision errors in dual-energy X-Ray absorptiometry measurements. J Clin Densitom jul.

[32] Evans EM, Mojtahedi MC, Kessinger RB, Misic MM (2006). Simulated change in body fatness affects Hologic QDR 4500A whole body and central DXA bone measures. J Clin Densitom.

[33] Bolotin HH (2007). DXA in vivo BMD methodology: an erroneous and misleading research and clinical gauge of bone mineral status, bone fragility, and bone remodelling. Bone.

[34] Meredith-Jones K, Haszard J, Stanger N, Taylor R (2018). Precision of DXA-Derived Visceral Fat measurements in a large sample of adults of varying body size. Obes Silver Spring Md mar.

[35] Gloria Bonaccorsi FP, Cafarelli C, Cervellati FrançoisD, Guio P, Greco M, Giganti (2020). Giuseppe Guglielmi a new corrective model to evaluate TBS in obese post-menopausal women: a cross-sectional study. Aging Clin Exp Res.

